# The prolonged impact of COVID-19 on symptoms, health-related quality of life, fatigue and mental well-being: a cross-sectional study

**DOI:** 10.3389/fepid.2023.1144707

**Published:** 2023-06-22

**Authors:** Iris M. Brus, Inge Spronk, Juanita A. Haagsma, Annemieke de Groot, Peter Tieleman, Sara Biere-Rafi, Suzanne Polinder

**Affiliations:** ^1^ Department of Public Health, Erasmus University Medical Center, Rotterdam, Netherlands; ^2^ C-support, ’s Hertogenbosch, Netherlands

**Keywords:** COVID-19, post COVID-19 condition, health-related quality of life (HRQL), fatigue, mental well-being, symptoms, SARS-CoV-2, long covid

## Abstract

**Background:**

A subset of patients experience persisting symptoms after an acute COVID-19 infection, referred to as “post COVID-19 condition”. This cross-sectional study aimed to compare symptoms, health-related quality of life (HRQoL), fatigue, mental well-being, and determinants of diminished HRQoL, between patients with post COVID-19 condition categorized by time since acute infection.

**Methods:**

We performed an online survey and analyzed responses of 10,194 adult respondents with a confirmed or suspected COVID-19 infection, who experienced persisting symptoms ≥3 months after the initial infection. The most debilitating symptoms and health outcomes were studied separately for respondents 3–6, 7–9, 10–12, 13–18, 19–24, and >24 months after acute infection.

**Results:**

At each time period, fatigue, sensory-processing problems, and concentration problems were the most debilitating symptoms reported by respondents, although the proportion of respondents who reported these symptoms differed significantly between time periods. Respondents 3–6 months post-acute infection had the lowest HRQoL (median EQ-5D utility score: 0.59), the highest fatigue level (median score: 110.0) and the highest proportion with a likely depressive disorder (32.4%), whereas respondents 13–18 months post-infection had the highest HRQoL (0.65), the lowest fatigue level (106.0), and the second lowest proportion with a likely depressive disorder (25.0%) (*p* = 0.000–0.007). Compared to those 13–18 and 19–24 months post-infection, respondents >24 months post-infection had a slightly lower HRQoL (0.60), lower fatigue level (108.0), and lower proportion with a likely depressive disorder (29.2%), although only the differences in HRQoL were statistically significant (*p* = 0.001–0.010). Younger age, female gender, lower level of education, not having paid work before COVID-19, comorbidity, and not being vaccinated, seemed to be associated with lower HRQoL.

**Conclusion:**

Regardless of time since infection, respondents considered fatigue, sensory processing problems and concentration problems the most debilitating symptoms. They experienced a low HRQoL and severe fatigue, even more than two years after acute COVID-19 infection. Respondents 3–6 months post-infection had the worst health outcomes, whereas respondents 13–18 months post-infection had the best outcomes, indicating that, at least for a subgroup of patients, health status may improve over time.

## 1. Introduction

Since the start of the COVID-19 pandemic in 2020, over 600 billion confirmed cases of COVID-19 have been reported worldwide (1). Although most patients recover from an acute COVID-19 infection, some experience long-lasting symptoms, usually referred to as “long COVID”, “post-COVID syndrome” or “post COVID-19 condition”, as defined by the World Health Organization (WHO) (2). Estimations of the proportion of individuals with post COVID-19 condition following acute infection vary from 4%–12% (3, 4). Due to the large number of people infected with COVID-19, post COVID-19 condition is a major public health challenge worldwide (5).

Post COVID-19 condition is usually defined as lasting symptoms at least three months after acute COVID-19 infection, as proposed by the WHO (2). However, previous studies used timeframes varying from four weeks to six months after acute infection (6). Post COVID-19 condition appears to affect patients across all disease severities, including those with a mild acute disease course, and across all age groups (3). Although there are currently many hypotheses regarding the cause of these lasting symptoms, the biological mechanism behind post COVID-19 condition has not yet been elucidated. Therefore, the broadly applied approach to improve these symptoms is rehabilitation, with no treatments having proven to be effective for patients with this disabling condition (6, 7).

Patients with post COVID-19 condition experience a wide range of symptoms, with fatigue, shortness of breath, concentration difficulties, and sleeping disorders being most frequently reported (8, 9). These symptoms can last for months or years after acute infection, and it is yet unknown whether they disappear in all patients over time. Possibly, these symptoms remain chronic in a subset of patients (10, 11). Post COVID-19 condition appears to have a significant effect on the mental well-being of patients, with previous research reporting increased rates of mood and anxiety disorders during the six months following a COVID-19 infection (12). Emerging evidence shows that these long-term symptoms after COVID-19 infection also have a negative impact on the health-related quality of life (HRQoL) of afflicted patients and affect patients' ability to function in daily life, including their ability to work (13, 14).

Many studies have investigated the symptoms that patients with post COVID-19 condition experience during the first months after acute infection. However, the nature and pattern of these long-term symptoms by time since acute infection, especially more than one year after acute infection, have not yet been fully clarified (10, 15, 16). Whether persistent symptoms remain, worsen or resolve over time, and to what extent post COVID-19 condition impacts HRQoL and mental well-being of afflicted patients is not yet fully known (15). Furthermore, there is a lack of knowledge on whether specific patients have an increased risk of long-lasting symptoms and diminished health outcomes (13). In addition, most studies have focused on the impact of post COVID-19 condition among hospitalized patients (8), while the majority of people infected with COVID-19 experience a mild acute disease course not requiring hospitalization (3).

A comprehensive overview of the impact of post COVID-19 condition on different dimensions of health and quality of life, as well as insight into observed patterns over time, is urgently needed to inform policymakers and improve healthcare and rehabilitation services (17). The aim of this cross-sectional study was therefore to compare symptoms, HRQoL, fatigue, mental well-being, and determinants of diminished HRQoL, between patients categorized by time since acute infection, in a large cohort of over 10,000 adult post COVID-19 patients.

## 2. Methods

### 2.1. Study design and respondents

The study was conducted in collaboration with C-support; a Dutch organization, commissioned by the Ministry of Health, that informs, advises and supports patients who experience long-term complaints after the initial COVID-19 infection. This cross-sectional study collected data via an online survey among patients with post COVID-19 condition that were registered at C-support. Between February 11 and November 16, 2022, a total of 18,074 patients of all ages who were registered at C-support were invited via email to complete the Dutch survey. Respondents were able to complete the online survey in steps, by saving their answers and resuming the survey later. If patients did not complete the survey, a reminder to complete the survey was sent after three weeks. Inclusion criteria for the present study were: adult patients (≥18 years old); a known infection date; and an infection date ≥3 months before completing the survey. All respondents provided online informed consent to use their data for scientific research. The Medical Ethics Review Board of the Erasmus Medical Center approved the study protocol (MEC-2021-0751).

### 2.2. Measures

#### 2.2.1. Socio-demographic and medical characteristics

The survey contained socio-demographic variables including age, gender, level of education, and living situation. Age was categorized into six groups: 18–24 years, 25–34 years, 35–44 years, 45–54 years, 55–64 years, 65–74 years and 75 years and older. Level of education was categorized as low (primary education, lower and middle general secondary education), middle (higher secondary education, middle vocational education) and high (higher professional education, university education) (18). Living situation was dichotomized as married or living with a significant other, and not married or living without a significant other.

Self-reported medical characteristics included month and year of COVID-19 infection, number of COVID-19 infections, hospitalization (yes/no) during acute infection, admission to Intensive Care Unit (yes/no), and vaccination status (vaccinated/not vaccinated). In addition, the survey included questions on height, weight and pre-existing chronic diseases. BMI was categorized as underweight (<18.5 kg/m^2^), normal weight (18.5–25 kg/m^2^) overweight (25–30 kg/m^2^) and obese (>30 kg/m^2^) (19). The list of pre-existing chronic diseases included 14 diseases and an option “other” (20).

#### 2.2.2. Symptoms

Based on a literature search, input from multiple patients living with post COVID-19 condition, and input from healthcare professionals, the occurrence of a total of 34 symptoms was assessed (Supplementary File S1, Table S2). Respondents specified which symptoms they had experienced since acute infection of COVID-19, and which of those symptoms they considered most debilitating during the last week. A maximum of five symptoms could be selected as most debilitating.

#### 2.2.3. Recovery from COVID-19

Recovery from COVID-19 was assessed using a question from the COVID-19-COS set of core outcome measures (21), as recommended by Munblit et al. (22). Respondents indicated to what extent they had recovered from COVID-19, with complete recovery meaning that respondents no longer had symptoms related to COVID-19, could do their usual daily activities and had returned to their previous state of health (prior to their COVID-19 illness). Response categories range from 1 (“not recovered at all”) to 5 (“completely recovered”).

#### 2.2.4. Health-related quality of life (HRQoL)

HRQoL was measured using the EQ-5D-5l (23), a generic instrument consisting of five items that each comprise one dimension of HRQoL: mobility, self-care, usual activities, pain/discomfort and anxiety/depression. Each item has five response categories: “no problems”, “slight problems”, “moderate problems”, “severe problems” and “extreme problems”. Using a Dutch value set (24), the responses were combined to compute a summary score (“utility index”), which is anchored from 1 (“full health”) to 0 (“death”). Respondents were also asked to score how they perceived their overall health status on a visual analogue (VAS) scale ranging from 0 (“worst imaginable health”) to 100 (“best imaginable health”).

#### 2.2.5. Fatigue, energy level, and post-exertional malaise

Fatigue was measured with the Checklist Individual Strength (CIS) (25). This validated questionnaire consists of 20 items on a 7-point Likert scale and assesses four different aspects of fatigue: fatigue severity (8 items), concentration problems (5 items), reduced motivation (4 items) and reduced activity level (3 items). Total scores range from 20 to 140, with higher scores indicating more fatigue. A score of 35 or more on the subscale fatigue severity is indicative of severe fatigue (26).

Respondents also indicated their energy level on average during the previous two weeks as a percentage, compared to the situation before COVID-19, with the initial situation being 100%.

Post-exertional malaise was described as follows: “Marked and rapid physical and/or cognitive fatigue, sometimes due to minimal effort. This fatigue can be debilitating and trigger a relapse” (27). Respondents rated the severity of post-exertional malaise on a 5-point scale: “no problems”, “slight problems”, “moderate problems”, “severe problems” and “extreme problems”.

#### 2.2.6. Anxiety and depression

Anxiety was measured using the short version of the Generalized Anxiety Disorder 7-item (28). The GAD-2 consists of two items assessing how often respondents were affected by each symptom during the last two weeks, with response categories ranging from 0 (“not at all”) to 3 (“nearly every day”). Total scores range from 0 to 6 and a score of 3 or higher is indicative of generalized anxiety disorder.

Depression was measured using the short version of the Patient Health Questionnaire 9-item (PHQ-2) (29), which, similar to the GAD-2, consists of two items with answers ranging from 0 (“not at all”) to 3 (“nearly every day”). A score of 3 or higher is indicative of major depressive disorder.

### 2.3. Statistical analyses

As there was large variation within our study population with regards to time since acute COVID-19 infection, and as time since infection appears to be an important factor in the recovery from post COVID-19 condition (30, 31), outcomes were studied separately for respondents who were at different time periods since (first) acute infection at the time of the survey. These different time periods included: 3–6 months (3–6 m), 7–9 months (7–9 m), 10–12 months (10–12 m), 13–18 months (13–18 m), 19–24 months (19–24 m), and >24 months (>24 m) since acute infection.

Descriptive statistics were performed to report sociodemographic and medical characteristics, symptoms, recovery from COVID-19, EQ-5D-5l utility score, EQ-VAS, EQ-5D-5l dimensions, GAD-2, PHQ-2, CIS summary score, CIS subscales, energy level and post-exertional malaise. For figures and regression analyses, the EQ-5D-5l, CIS sum score and CIS subscales were transformed to a 0–100 scale, with higher scores indicating better health, in order to be comparable with other variables. Continuous data were reported as mean and standard deviation (SD) if normally distributed, and as median and interquartile range (IQR) if not-normally distributed. Categorical data were reported as numbers (percentage). Continuous variables were compared between the different time periods since acute infection with ANOVA tests if normally distributed, or with Kruskal-Wallis tests if not normally distributed, and categorical variables were compared using Chi-square tests. When the test result was significant (*p*-value <0.05) for the comparison of a variable, Bonferroni-corrected pairwise comparisons were conducted to determine differences.

To identify determinants of diminished HRQoL, linear regression analyses were performed for the EQ-5D utility score and the EQ VAS using sociodemographic and medical characteristics as independent variables. Variables with a *p*-value <0.10 in univariate analyses were checked for multicollinearity [variance inflation factors (VIF) < 5] and included in multivariate regression analyses (32). A *p*-value of <0.05 was considered statistically significant. All analyses were performed using IBM SPSS version 28.

## 3. Results

### 3.1. Characteristics of respondents

A total of 18,074 patients registered at C-support were invited to participate, of whom 10,385 completed the survey (57.5%). Of those, 191 respondents were excluded because they were either ≤17 years old (*n* = 75), had a missing date of acute infection (*n* = 8) or were infected <3 months before completing the survey (*n* = 108). Therefore, a total of 10,194 respondents were included.

Time since (first) acute infection varied between respondents, ranging from 3 to 6 months (16.7% of respondents) to >24 months (5.2%) prior to completing the survey (Table 1). Table 1 describes the characteristics of respondents for the different groups by time period since acute infection. The median age was 45.0 (IQR = 18.0) for those 3–6 m post-infection, and 52.0 (IQR = 17.0) for those >24 m post-infection, with more recently infected respondents being younger (*p* < 0.001). The majority was female (65.8–79.5%), with those >24 m post-infection having a significantly lower percentage of females than all other groups (*p* < 0.02). Most respondents had a high level of education (50.3%–58.3%), had paid work before COVID-19 (87.5%–93.1%), and were married or living with a significant other (68.5%–76.1%). More recently infected respondents (3–6 m) had a significantly higher proportion with a high level of education than those 10–12 m, 13–18 m, 19–24 m, and >24 m post-infection (*p* < 0.001). They also had a significantly higher proportion with paid work before COVID-19 than those 19–24 m and >24 m post-infection (*p* < 0.01). Comorbidity only significantly differed between those 19–24 m post-infection and those 3–6 m, 10–12 m, and 13–18 m post-infection (*p* < 0.01), with more recently infected respondents having a lower proportion with comorbidity. Three to ten percent of respondents were admitted to a hospital, with those 3–6 m post-infection having the lowest admission rate (2.6%) (*p* < 0.01). Intensive care admission did not differ significantly between respondents 3–6 m, 7–9 m, 10–12 m, 13–18 m, 19–24 m, and >24 m post-infection. Most respondents (90.8–93.3%) were vaccinated at the time of the survey, although only 25.4% was vaccinated before the first acute COVID-19 infection.

**Table 1:** Characteristics of respondents.

	Time since acute infection						*p*-Value
	3–6 months	7–9 months	10–12 months	13–18 months	19–24 months	>24 months	
	*n* = 1,705	*n* = 1,210	*n* = 1,354	*n* = 2,785	*n* = 2,613	*n* = 527	
**Age in years, median (IQR)**	45.0 (18.0)	46.0 (18.0)	48.0 (17.0)	49.0 (16.0)	51.0 (15.0)	52.0 (17.0)	
**Age in categories, *n* (%)**							<0.001
18–24 years	56 (3.3)	45 (3.7)	38 (2.8)	71 (2.5)	37 (1.4)	10 (1.9)	
25–34 years	306 (17.9)	211 (17.4)	202 (14.9)	354 (12.7)	245 (9.4)	55 (10.4)	
35–44 years	448 (26.3)	281 (23.2)	284 (21.0)	593 (21.3)	505 (19.3)	91 (17.3)	
45–54 years	503 (29.5)	389 (32.1)	430 (31.8)	932 (33.5)	841 (32.2)	156 (29.6)	
55–64 years	332 (19.5)	250 (20.7)	343 (25.3)	723 (26.0)	812 (31.1)	167 (31.7)	
65–74 years	56 (3.3)	30 (2.5)	50 (3.7)	92 (3.3)	150 (5.7)	41 (7.8)	
75–88 years	4 (0.2)	4 (0.3)	7 (0.5)	20 (0.7)	23 (0.9)	7 (1.3)	
**Gender, *n* (%)**							<0.001
Male	343 (20.1)	287 (23.7)	346 (25.6)	650 (23.3)	631 (24.1)	177 (33.6)	
Female	1,356 (79.5)	918 (75.9)	1,008 (74.4)	2,130 (76.5)	1,974 (75.5)	347 (65.8)	
Other	4 (0.2)	4 (0.3)	-	3 (0.1)	5 (0.2)	2 (0.4)	
Rather not disclose	2 (0.1)	1 (0.1)	-	2 (0.1)	3 (0.1)	1 (0.2)	
**Level of education, *n* (%)**							<0.001
Low	155 (9.1)	142 (11.7)	186 (13.7)	346 (12.4)	325 (12.4)	87 (16.5)	
Middle	554 (32.5)	390 (32.2)	454 (33.5)	1,004 (36.1)	928 (35.5)	174 (33.0)	
High	994 (58.3)	673 (55.6)	708 (52.3)	1,430 (51.3)	1,357 (51.9)	265 (50.3)	
Unknown	2 (0.1)	5 (0.4)	6 (0.4)	5 (0.2)	3 (0.1)	1 (0.2)	
**Occupational status before COVID-19, *n* (%)**							<0.001
Paid work	1,586 (93.0)	1,126 (93.1)	1,246 (92.0)	2,550 (91.6)	2,337 (89.4)	461 (87.5)	
Unpaid work	34 (2.0)	10 (0.8)	18 (1.3)	44 (1.6)	78 (3.0)	20 (3.8)	
No work	85 (5.0)	74 (6.1)	90 (6.6)	191 (6.9)	198 (7.6)	46 (8.7)	
**Married/living with significant other, *n* (%)**							<0.001
Yes	1,210 (71.0)	870 (71.9)	1,030 (76.1)	2,050 (73.6)	1,815 (69.5)	361 (68.5)	
No	493 (28.9)	339 (28.0)	320 (23.6)	727 (26.1)	794 (30.4)	166 (31.5)	
Unknown	2 (0.1)	1 (0.1)	4 (0.3)	8 (0.3)	4 (0.2)	-	
**Comorbidity, *n* (%)**							<0.001
No comorbidity	930 (54.5)	653 (54.0)	750 (55.4)	1,506 (54.1)	1,281 (49.0)	253 (48.0)	
Comorbidity	775 (45.5)	557 (46.0)	604 (44.6)	1,279 (45.9)	1,332 (51.0)	274 (52.0)	
**BMI, *n* (%)**							<0.001
Underweight (<18.5 kg/m^2^)	22 (1.3)	20 (1.7)	20 (1.5)	37 (1.3)	37 (1.4)	7 (1.3)	
Normal weight (18.5–25 kg/m^2^)	828 (48.6)	518 (42.8)	501 (37.0)	1,063 (38.2)	1,029 (39.4)	209 (39.7)	
Overweight (25–30 kg/m^2^)	519 (30.5)	394 (32.6)	477 (35.2)	976 (35.0)	955 (36.5)	175 (33.2)	
Obese (>30 kg/m^2^)	334 (19.6)	278 (23.0)	355 (26.2)	709 (25.5)	590 (22.6)	134 (25.4)	
Unknown	2 (0.1)	-	1 (0.1)	-	2 (0.1)	2 (0.4)	
**COVID-19 diagnosis, *n* (%)**							<0.001
Confirmed with test	1,682 (98.7)	1,195 (98.8)	1,322 (97.6)	2,725 (97.8)	1,554 (59.5)	363 (68.9)	
Not confirmed with test	23 (1.3)	15 (1.2)	32 (2.4)	60 (2.2)	1,059 (40.5)	164 (31.1)	
**Number of COVID-19 infections, *n* (%)**							<0.001
1 infection	1,552 (91.0)	986 (81.5)	1,068 (78.9)	2,115 (75.9)	1,791 (68.5)	178 (33.8)	
2 or more infections	153 (9.0)	224 (18.5)	286 (21.1)	670 (24.1)	822 (31.5)	349 (66.2)	
**Hospital admission, *n* (%)**							<0.001
Yes	45 (2.6)	78 (6.4)	140 (10.3)	270 (9.7)	273 (10.4)	42 (8.0)	
No	1,660 (97.4)	1,132 (93.6)	1,214 (89.7)	2,515 (90.3)	2,340 (89.6)	485 (92.0)	
**Intensive care admission, *n* (%)**							0.091
Yes	7 (0.4)	16 (1.3)	45 (3.3)	75 (2.7)	70 (2.7)	16 (3.0)	
No	1,698 (99.6)	1,194 (98.7)	1,309 (96.7)	2,710 (97.3)	2,543 (97.3)	511 (97.0)	
**Vaccination status, *n* (%)**							0.003
Vaccinated	1,552 (91.0)	1,097 (90.7)	1,229 (90.8)	2,599 (93.3)	2,433 (93.1)	479 (90.9)	
Not vaccinated	132 (7.7)	97 (8.0)	112 (8.3)	163 (5.9)	158 (6.0)	44 (8.3)	
Rather not disclose	21 (1.2)	16 (1.3)	13 (1.0)	23 (0.8)	22 (0.8)	4 (0.8)	

This table shows the characteristics of respondents, presented separately for respondents at different time periods since acute infection.

### 3.2. Top 10 most debilitating symptoms

**Figure 1:**
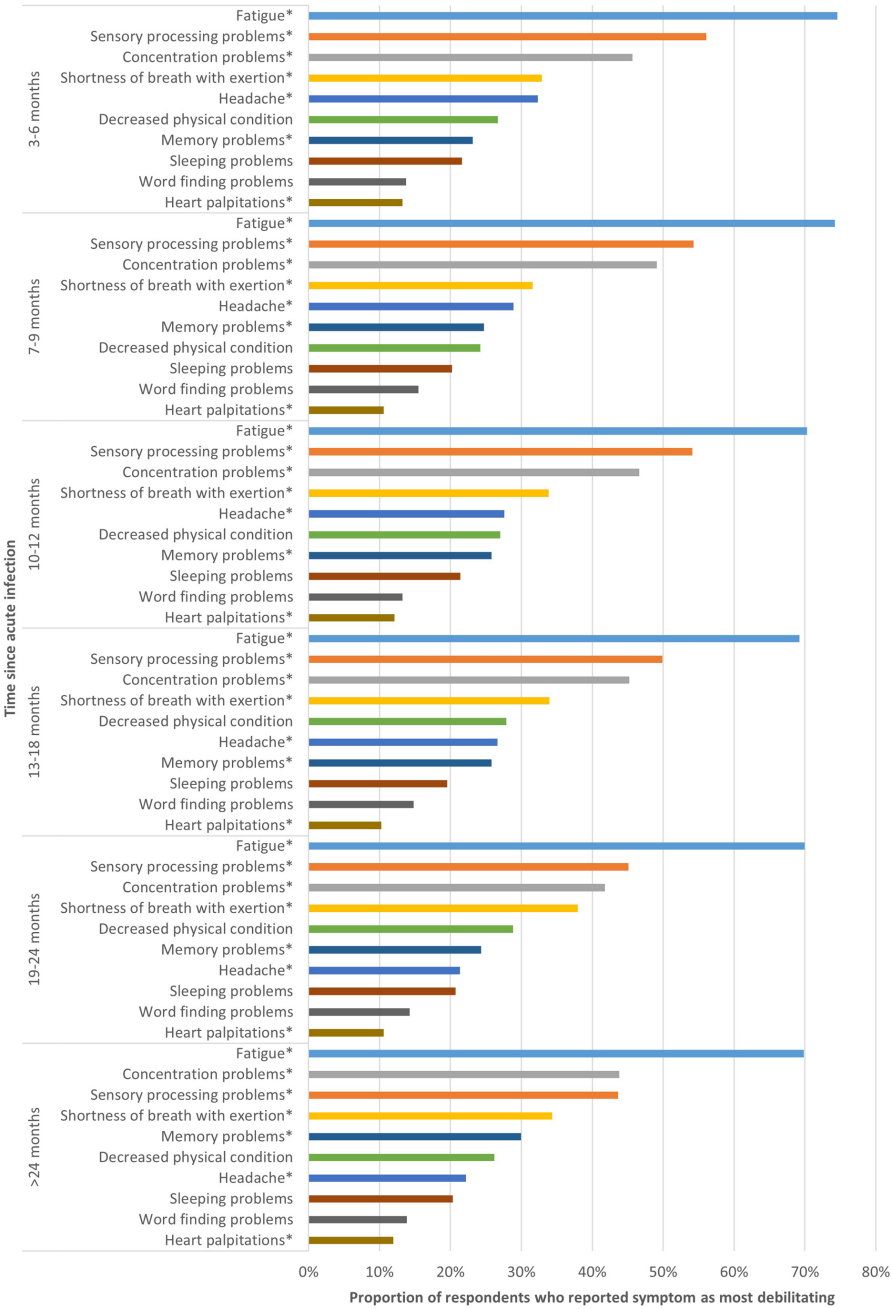
Top 10 most debilitating symptoms per time period. This figure shows the top 10 symptoms respondents considered most debilitating, presented separately for respondents at different time periods since acute infection. *Statistically significant differences (*p* < 0.05) between groups based on time since acute COVID-19 infection.

For each time period, respondents indicated that fatigue, sensory processing problems and concentration problems were the most debilitating symptoms (Figure 1). However, the proportion of respondents reporting these symptoms as most debilitating differed among the studied time periods. Of those 3–6 m post-infection, 74.5% indicated fatigue as most debilitating, which was significantly higher than those 13–18 m (69.2%) and 19–24 m post-infection (70.0%) (*p* < 0.02). The proportion of respondents that indicated concentration problems as most debilitating differed significantly between those 7–9 m (49.1%) and 19–24 m post-infection (41.8%) (*p* < 0.001).

Some symptoms were significantly more often reported as most debilitating by those most recently infected (3–6 m) compared to those with the longest disease duration (>24 m), such as sensory processing problems (56.1% vs. 43.6%; *p* < 0.001) and headache (32.3% vs. 22.2%; *p* < 0.001).

In contrast, other symptoms were less frequently reported as most debilitating by those most recently infected, such as shortness of breath with exertion, for which there was significant difference between those 3–6 m (32.9%) and 7–9 m post-infection (31.6%) compared to those 19–24 m post-infection (38.0%) (*p* < 0.01). Memory problems were significantly more frequently reported by those >24 m post-infection compared to those 3–6 m post-infection (23.2% vs. 30.0%; *p* = 0.024).

For decreased physical condition, sleeping problems and word finding problems, no significant difference between the six different time periods was found. The proportion of respondents indicating heart palpitations as most debilitating was only significantly different between those 3–6 m post-infection and those 13–18 m post-infection (13.3% vs. 10.2%; *p* = 0.028).

Other symptoms are shown in Supplementary File S1, Table S2.

### 3.3. Recovery from COVID-19

**Figure 2:**
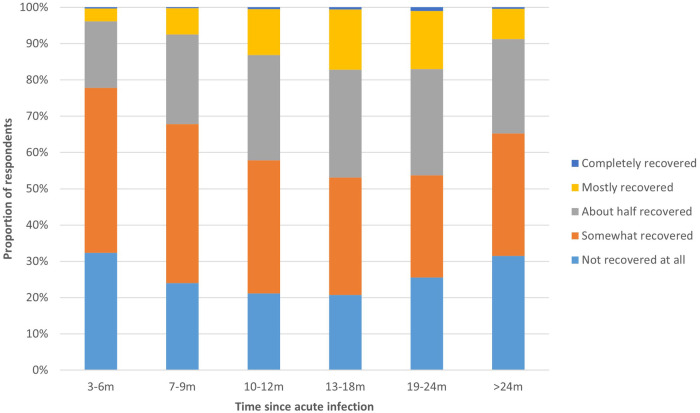
Recovery phase from COVID-19 per time period. This figure shows the extent to which respondents had recovered from COVID-19, presented separately for respondents at different time periods since acute infection.

The proportion of respondents in different phases of recovery from COVID-19 (not recovered to completely recovered) differed significantly between the time periods since acute infection (Figure 2). Of those 3–6 m post-infection, 77.8% indicated that they had not or only somewhat recovered, which was significantly higher compared to those with a longer disease duration (>6 months; *p* < 0.01). The proportion of respondents that indicated they had mostly or completely recovered increased from 3.9% among respondents 3–6 m post-infection to 17.0% among respondents 19–24 m post-infection. Among respondents >24 post-infection, 8.7% was mostly or completely.

### 3.4. Health outcomes

**Figure 3:**
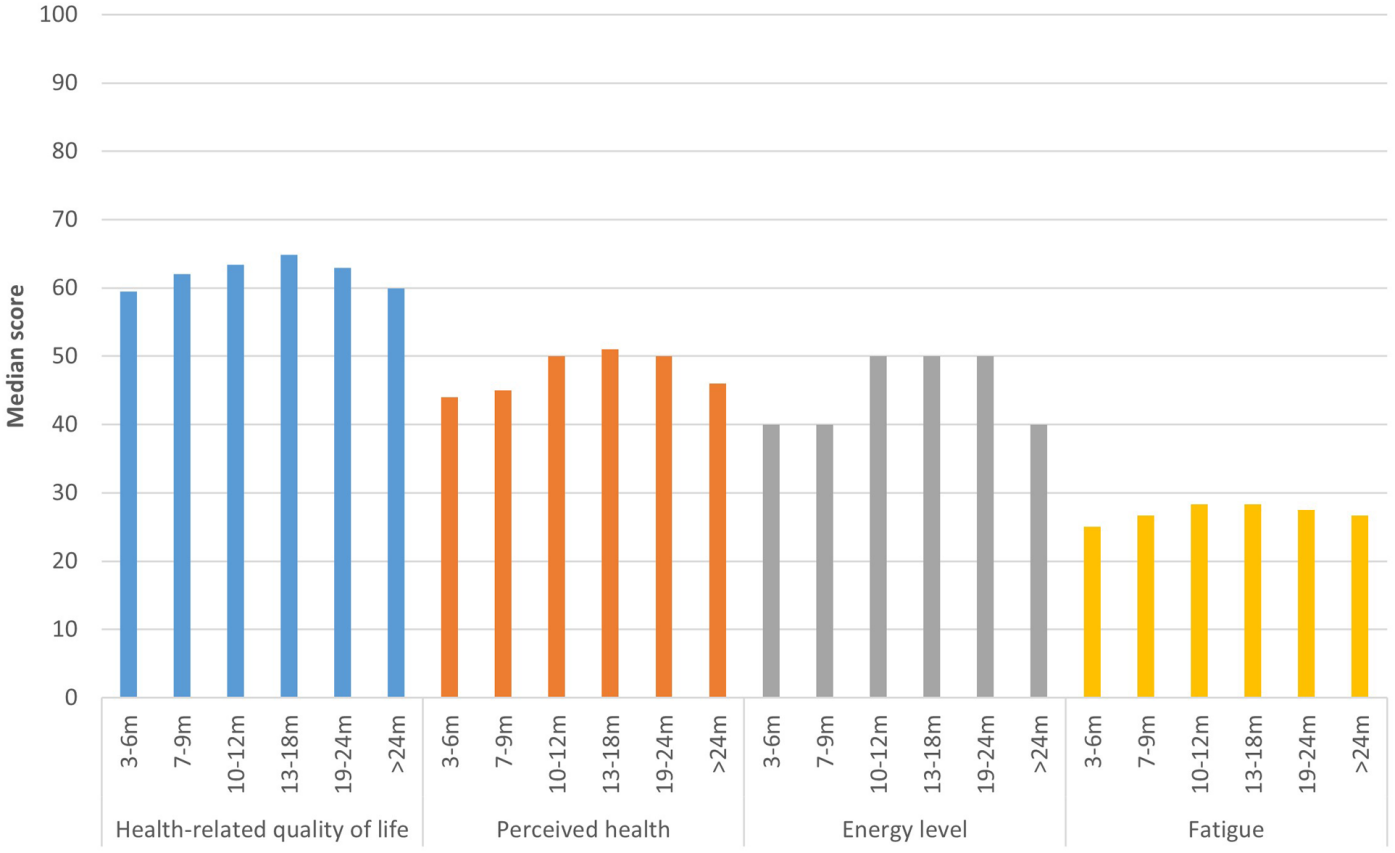
Health outcomes per time period. This figure shows the median health-related quality of life (EQ-5D utility score)*, perceived health status (EQ VAS), fatigue (CIS sum score)* and energy level compared to before COVID-19 of respondents, presented separately for respondents at different time periods since acute infection. *EQ-5D utility score and CIS sum score were transformed to a 0-100 scale, with higher scores indicated higher quality of life and less fatigue, in order to be comparable to other outcomes.

All health outcomes studied, HRQoL, perceived health, energy level, fatigue, post-exertional malaise, anxiety, and depression, differed significantly between time periods since acute infection (*p* < 0.001). Respondents 3–6 m post-infection had the lowest HRQoL (median EQ-5D utility score: 0.59, IQR = 0.32; median EQ VAS: 44.0, IQR = 30.0), whereas those 13–18 m post-infection had the best HRQoL (median EQ-5D utility score: 0.65, IQR = 0.33; median EQ VAS: 51.0, IQR = 29.0) (Figure 3; Supplementary File S1, Table S2). EQ-5D-5l dimensions usual activities and pain/discomfort were most affected, with >50% of respondents experiencing moderate to extreme problems on these dimensions. There were significant differences between time periods for all dimensions (*p* < 0.001), except for anxiety/depression (*p* = 0.11) (Supplementary File S1, Figure S1).

**Figure 4:**
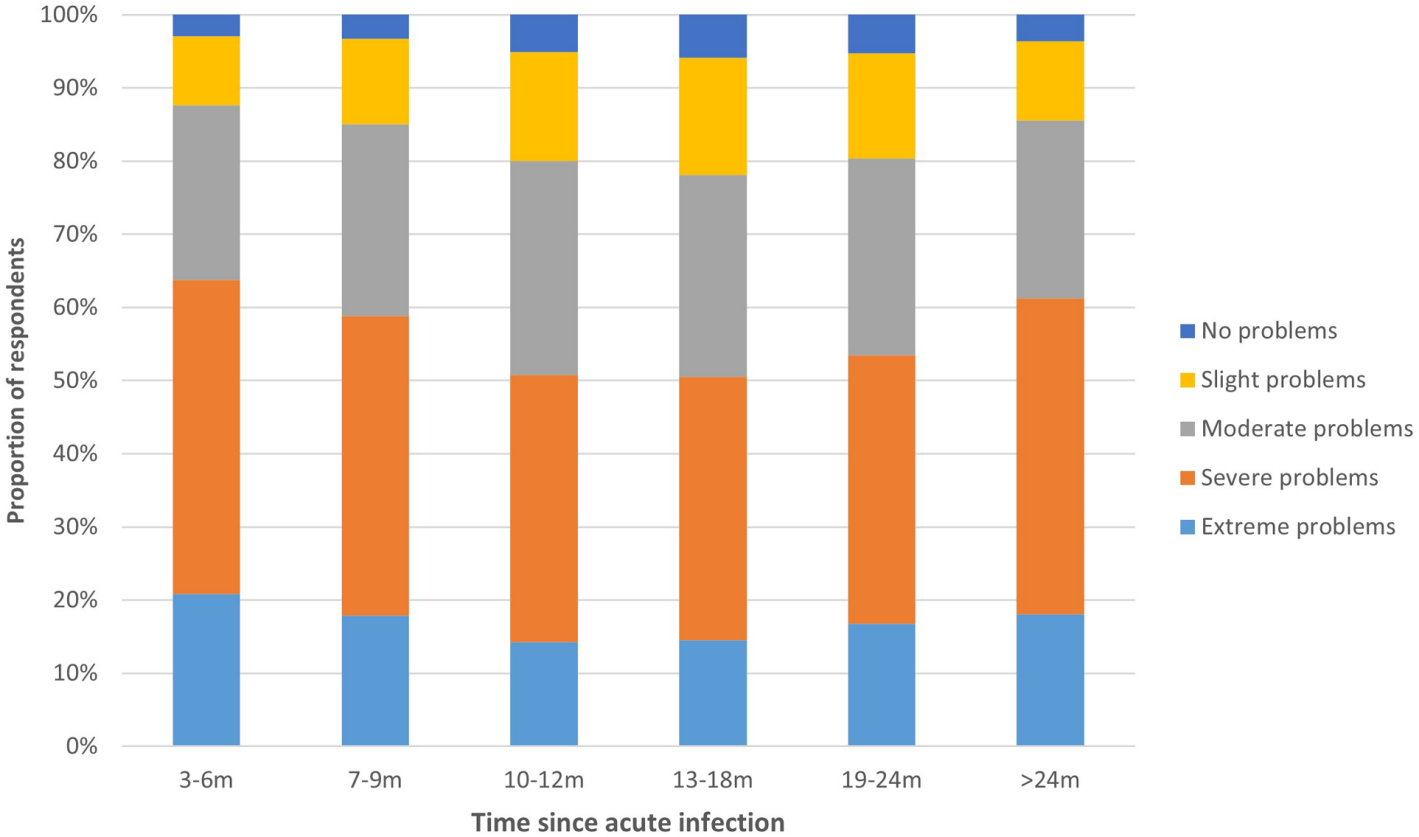
Post-exertional malaise per time period. This figure shows the degree to which respondents experience problems with post-exertional malaise, presented separately for respondents at different time periods since acute infection.

Median energy level compared to before COVID-19 was 40.0% (IQR = 25.0–30.0) for those 3–6 m, 7–9 m, and >24 m post-infection, and 50.0% (IQR = 31.3–35.0) for those 10–12 m, 13–18 m and 19–24 m post-infection (Figure 3). Fatigue was highest for those 3–6 m post-infection (median CIS sum score: 110.0, IQR = 23.0), lowest for those 13–18 m post-infection (106.0, IQR = 26.0), and those >24 m post-infection reported a fatigue score in between (108.0, IQR = 25.0). There were significant differences between time periods for all dimensions of fatigue (*p* < 0.001), except reduced motivation (*p* = 0.771) (Supplementary File S1, Figure S2). The majority of respondents experienced severe or extreme symptoms of post-exertional malaise, with those 3–6 m post-infection being most frequently affected (63.8%) compared to 50.5% of those 13–18 m and 61.3% of those >24 m post-infection (Figure 4).

**Figure 5:**
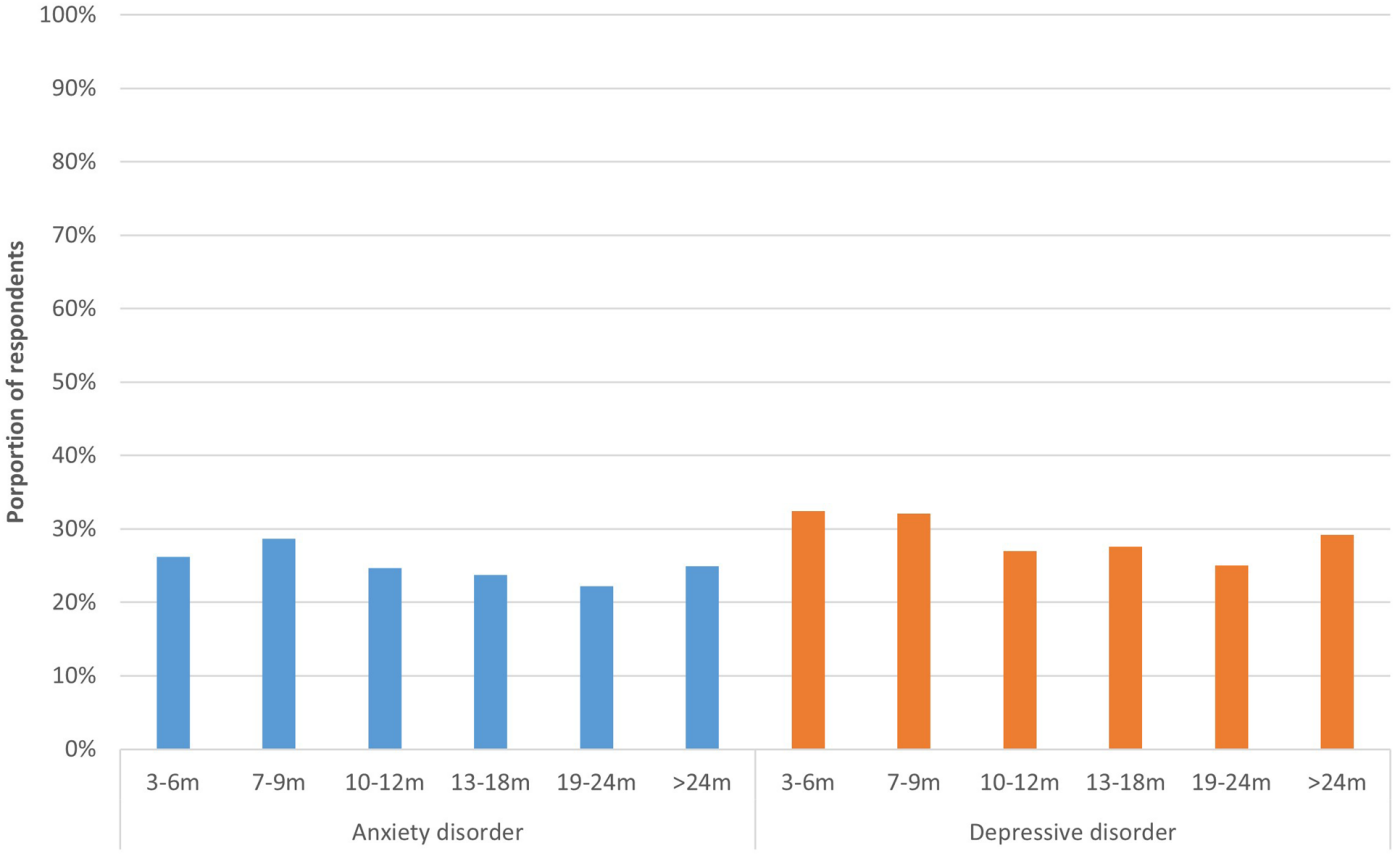
Anxiety and depression per time period. This figure shows the proportion of respondents who have a score (≥3) indicative of general anxiety disorder (PHQ-2) and depressive disorder (GAD-2), presented separately for respondents at different time periods since acute infection.

A total of 26.2% of respondents 3–6 m post-infection had a score indicative of anxiety disorder and 32.4% had a score indicative of depressive disorder (Figure 5). For those 19–24 m post-infection, these proportions were lowest, 22.2% and 25.0% respectively, and they were significantly different from those infected most recently (respectively *p* < 0.042 and *p* < 0.001).

### 3.5. Determinants of HRQoL

Multivariate regression analyses showed that age seemed to be associated with worse HRQoL (Table 2). Only for those 10–12 m post-infection we found no association between age and EQ-5D utility scores. Female gender seemed to be associated with worse utility scores for all time periods, except for those >24 m post-infection, and with worse EQ VAS scores for those 3–6 m and 19–24 m post-infection. A lower level of education seemed to be associated with worse utility scores for all time periods, and with worse EQ VAS scores for those 7–9 m post-infection. Not having paid work before COVID-19 seemed to be associated with lower utility scores for all time periods, and with worse EQ VAS scores for those 13–18 m post-infection.

**Table 2:** Multivariate linear regression analyses for EQ-5D-5l utility score (transformed) and EQ-VAS.

	EQ-5D utility scores (transformed)						EQ VAS					
	3–6 months	7–9 months	10–12 months	13–18 months	19–24 months	>24 months	3–6 months	7–9 months	10–12 months	13–18 months	19–24 months	>24 months
	*n* = 1,672	*n* = 1,183	*n* = 1,333	*n* = 2,745	*n* = 2,574	*n* = 517	*n* = 1,672	*n* = 1,183	*n* = 1,333	*n* = 2,745	*n* = 2,574	*n* = 517
**Age**												
18–24 years	−7.732*	−5.989		−2.486	−10.607*	2.522	−1.462	−2.490	−1.256	−4.841*	−8.287*	−5.516
25–34 years	−5.621*	−6.305*		−4.010*	−5.538*	−6.052	−2.785*	−3.903*	0.206	−3.155*	−2.769*	−0.386
35–44 years	−1.993	−6.511*		−1.650	−3.256*	−1.183	−1.381	−3.059*	−0.433	−1.407	−1.403	1.046
45–54 years (ref)												
55–64 years	1.382	−2.960		0.151	1.022	4.844	1.431	0.044	2.757*	0.609	1.912*	5.064*
65–88 years	5.918	9.759*		11.055*	8.716*	15.802*	2.707	11.739*	5.650*	5.098*	8.859*	8.889*
**Gender**												
Male	5.094*	5.595*	5.117*	3.826*	6.364*		2.919*	1.808	1.583	1.627	3.324*	
Female (ref)												
**Level of education**												
Low	−1.209	−5.074*	−7.791*	−7.108*	−6.542*	−7.685*	0.465	−3.700*				
Middle	−3.481*	−5.147*	−3.825*	−2.426*	−2.468*	−2.837	−1.600	−2.666*				
High (ref)												
**Married/living with significant other**												
Yes (ref)												
No	−1.778	−3.137		−1.161	−2.513*		−1.382			−1.312	−1.734*	
**Occupational status before COVID-19**												
Paid work (ref)												
No paid work	−9.522*	−6.556*	−9.878*	−9.879*	−5.129*	−8.196*				−3.266*		
**Comorbidity**												
Yes	−5.406*	−5.713*	−5.609*	−6.460*	−7.971*	−9.203*	−3.805*	−2.632*	−4.058*	−3.713*	−4.802*	−5.192*
No (ref)												
**Overweight**												
Yes (ref)												
No				0.636	3.867*	7.638*					2.169*	4.211*
**Hospital admission**												
Yes				−2.225			5.454*					5.604
No (ref)												
**Vaccinated**												
Yes (ref)												
No		−6.379*	−7.671*	−2.734	−5.764*				−5.352*	−2.505	−5.388*	

This table shows the results of the multivariate linear regression analyses, presented separately for respondents at different time periods since acute infection. Respondents with missing data on gender, level of education, household situation (married/living with significant other), or BMI, were excluded from the regression analyses (*n* = 170; 1.7%). EQ-5D utility scores were transformed to a 0–100 scale.

* Statistically significant determinants (*p* < 0.05).

Comorbidity seemed to be associated with lower HRQoL (lower utility and EQ VAS scores) for all time periods, with regression coefficients being higher for those with a longer disease duration. Regression coefficients for utility scores were −5.4 (*p* < 0.001) for those 3–6 m post-infection, −6.5 (*p* < 0.001) for those 13–18 m post-infection, and −9.2 (*p* < 0.001) for those >24 m post-infection. Being overweight seemed to only be associated with worse utility and EQ VAS scores for those 19–24 and >24 m post-infection. Lastly, not being vaccinated seemed to be associated with worse utility scores for those 7–9 m, 10–12 m and 19–24 m post-infection, and with worse EQ VAS scores for those 10–12 m and 19–24 m post-infection.

## 4. Discussion

This study compared symptoms, HRQoL, fatigue, mental well-being, and determinants of diminished HRQoL, between patients with post COVID-19 condition categorized by time since acute infection. We found that, at each time period, respondents reported similar symptoms (e.g., fatigue, sensory processing problems) as most debilitating, although they were more frequently reported by more recently infected respondents. Furthermore, we found that respondents experienced a low HRQoL and severe fatigue, even more than two years after acute COVID-19 infection. At each time period, 22%–32% had a score indicative of anxiety or depressive disorder. Respondents 3–6 m post-infection had the worst long-term health outcomes (HRQoL, fatigue, anxiety, depression), whereas those 12–18 m post-infection had the best health outcomes.

Respondents reported a wide range of symptoms, with fatigue, sensory processing problems, concentration problems, and shortness of breath with exertion being reported as the most debilitating symptoms for each time period. These results are confirmed by a large population-based, cross-sectional study in Germany that found that fatigue, neurocognitive impairment, and chest symptoms, such as shortness of breath, contributed most to reduced health recovery and working capacity (33). Our results showed that several symptoms, such as fatigue, sensory processing problems and headache, were more frequently reported as most debilitating by recently infected respondents, which may indicate that these symptoms decrease over time. In contrast, symptoms such as shortness of breath with exertion and memory problems were more often reported by those with a longer disease duration (19–24 m and >24 m post-infection), although these patterns were not completely clear, possible due to our cross-sectional design. For other symptoms, such as decreased physical condition and sleeping problems, we found no difference between those at different time points post-infection, implying that these symptoms may not improve over time.

In previous studies with a longitudinal design, several different symptom patterns over time were observed. Tran et al. found that fatigue and headache, but also memory problems, dyspnea and sleeping problems decreased over time (31). A systematic review by Yang et al. showed that most post COVID-19 symptoms decreased after 9 months, although fatigue persisted (30). However, these studies only had a follow-up period of 12 months. A longitudinal study with a 2 year follow-up among hospitalized patients found that, while the proportion of patients that experienced symptoms such as fatigue or muscle weakness, sleep difficulties and dizziness decreased between 6 and 12 months, it actually significantly increased between 12 months to 2 years (34). Although our results are not directly comparable to these studies due to the difference in study design, our data similarly suggests that symptoms might fluctuate over time, rather than follow a linear course. These fluctuating patterns indicate the need for studies with a longer follow-up duration to investigate whether this relapsing course eventually improves or whether symptoms become chronic.

The possible decrease in some symptoms raises the question whether these symptoms resolve over time, or whether patients develop certain coping strategies, adjusting their activities in response to their symptoms. For example, sensory processing problems appeared to be more debilitating for those recently infected, which is also observed in other studies (35). This symptom is strongly related to the physical situation the patient is in (e.g., crowds, loud music), which might lead to patients avoiding these situations, resulting in a reported decrease in symptoms, even if the symptom actually remains. Previous research shows that chronic diseases, e.g., chronic pain, can lead to avoidance behavior, which is often beneficial, but can also result in a decrease in functional ability (36). Thus, future research should investigate whether patients develop coping strategies to better deal with post COVID-19 condition symptoms, and the effect of these strategies on long-term recovery.

Respondents reported a substantially lower HRQoL compared to the general Dutch population (mean EQ-5D index: 0.52–0.58 vs. 0.89; mean EQ VAS: 44.2–50.2 vs. 82.0) (37).The HRQoL observed in our study is also considerably lower than in a previous systematic review, which reported scores close to the Dutch population norms (mean EQ VAS: 81.1) (13). This may indicate that our study sample was not representative of all post COVID-19 patients, or not comparable to the study populations included in the systematic review, possibly due to selection and/or non-response bias. Patients mostly or fully recovered from post COVID-19 condition could either not be registered in the C-support post COVID-19 condition registry or be less inclined to participate in this study, resulting in a specific subset of patients with more severe symptoms that filled out our survey. This might especially be the case for those with a longer disease duration, as the proportion of respondents that indicated to have mostly of fully recovered was significantly lower for those >24 m post-infection compared to those 13–18 m and 19–24 m post-infection.

Our results showed that the EQ-5D dimensions usual activities and pain/discomfort were most affected, with >50% of respondents reporting moderate to extreme problems. This is extremely high compared to the general Dutch population, in which only 14% reported problems with usual activities, and 34% with pain/discomfort (37). The energy level of respondents was <50% compared to before COVID-19 and fatigue scores were substantially higher than in the general population (mean CIS sum score: 103.1–107.3 vs. 54.8) (26). Median scores on the subscale fatigue severity in our study were similar to those found in a follow-up study among post COVID patients in Germany (47.0–50.0 vs. 46.0–48.0) (38). The majority of respondents in our study (51%–64%) experienced severe to extreme post-exertional malaise, which is in line with previous cross-sectional research that concluded that 59% of post COVID patients met the post-exertional malaise scoring thresholds used in people living with myalgic encephalomyelitis/chronic fatigue syndrome (39). Post COVID-19 condition also appeared to affect mental well-being, as about a quarter of respondents had a score indicative of anxiety disorder or depressive disorder. This seems to be high compared to the general population: depending on age group, 3.9%–8.9% of the Dutch population has a high risk of an anxiety or depressive disorder (40). However, these rates do seem to be comparable to previous studies among patients with post COVID-19 condition, although these estimates vary widely (41).

All health outcomes were worst for respondents 3–6 m post-infection, and best for those 13–18 m post-infection, which seems to imply that, at least for a proportion of respondents, health status improves over time. Although this again raises the question whether outcomes actually improve, or whether patients adjust their life style and/or their mindset to the new situation, and adapt to the experienced complaints, as previous research among patients with a variety of chronic diseases observed adaptations in multiple life domains (36, 42). Interestingly, for respondents >24 m post-infection, health outcomes were worse than for those 13–18 and 19–24 m post-infection. In a study with a longitudinal design, Tran et al. observed a similar U-shaped trend in the development of the perception of the impact of post COVID-19 condition on patients' lives (31), although they found an aggravation 6 months after onset. Tran et al. theorized that the aggravation relates to the realization that persisting symptoms might be chronic rather than temporary. Although this could play a role, as our design is cross-sectional, we hypothesize that the U-shaped pattern observed in our results is at least partly due to the previously mentioned selection and/or non-response bias. Another possible explanation could be that respondents >24 m post-infection were infected with a different COVID-19 virus variant leading to more severe long-term complaints. Recent studies indicate that the prevalence, nature and severity of post COVID-19 condition might differ depending on the COVID-19 virus variant (43, 44). In addition, previous research also suggests that COVID-19 vaccination might impact post COVID-19 condition, and those infected in early stages of the pandemic were less likely to be vaccinated before infection (45). Longitudinal studies with a follow-up duration of multiple years could provide more insight into the recovery from post COVID-19 condition over time, although such studies also put a significant burden on patients that already experience severe fatigue and neurocognitive symptoms, and run a similar risk of loss to follow-up of recovered patients with few complaints.

Although we observed statistically significant differences for several symptoms, and all other studied health outcomes between the different time periods since acute infection, these differences were relatively small: e.g., the EQ-5D index score ranged from 0.52–0.58 and the EQ VAS from 44.4–50.2. Whether these could be considered clinically important differences for post COVID-19 condition remains unclear, as estimates of minimum clinically important differences vary (46). However, as we hypothesize that (mostly) recovered patients were less likely to participate in our study, especially those at longer time periods since acute infection, the actual differences in health outcomes between patients at different time periods might be more pronounced, and underestimated in this study.

Our results showed that, regardless of time since infection, younger age, female gender, lower level of education, not having paid work before COVID-19, comorbidity, and not being vaccinated, appear to be associated with worse HRQoL. This is in line with previous research investigating the risk factors of developing post COVID-19 condition (47). Determinants were similar for those at different time points post-infection, which seems to indicate that determinants are largely independent of disease duration. However, comorbidity and being overweight were more important determinants for those with a longer disease duration, implying that general health status prior to COVID-19 might be associated with the speed of recovery from post COVID-19 condition. Investigating the impact of lifestyle changes among these patients is therefore a relevant avenue for future research, as this could possibly affect recovery.

Interestingly, although previous research suggests that the risk of post COVID-19 condition is greater in patients with a more severe acute disease course, especially those who need hospitalization (3), we found no association between hospital admission and HRQoL (EQ-5D). A possible explanation is that our study population is not a representative sample of patients who needed hospitalization, or that patients who would normally be hospitalized remained at home due to lower healthcare access and a lack of capacity during COVID-19 peaks (48). However, it could also be that hospitalized patients that experience long-term symptoms receive earlier and more extensive treatment than non-hospitalized patients, as rehabilitation is more accessible and healthcare professionals might be more aware of the possibility of lasting complaints among the former group. A previous cohort study found that patients who received rehabilitation initially had worse outcomes, but reached a similar level of physical function at 12 months follow-up as those who did not receive this care, indicating the impact early care and rehabilitation can have on recovery (49).

Earlier studies indicated some additional determinants that might be associated with worse health outcomes, such as ethnicity and smoking status, which we could not take into account in our analyses as items on these characteristics were not included in the survey (47). Furthermore, the influence of factors such as social support and self-efficacy also deserves attention, as previous research shows that these factors affect quality of life and other health outcomes in patients with chronic diseases, such as diabetes (36, 50).

The major strengths of our study include the large population of patients with post COVID-19 condition, as well as the wide range of symptoms and health outcomes that were studied, providing a comprehensive overview of the long-term impact of COVID-19. In addition, the response rate of 58% was relatively high, especially when taking into account the severe fatigue and neurocognitive symptoms respondents experienced (51).

This study has several limitations. First, as this study had a cross-sectional design, we cannot draw any definite conclusions about the development of post COVID-19 condition over time. However, due to the large number of respondents at each time period, an indication of possible patterns could be inferred from our data. A second limitation is the selection bias and non-response bias that were likely introduced, especially at longer time periods since infection. It appears that patients mostly or fully recovered from post COVID-19 condition, particularly 1.5–2 years after acute infection, are underrepresented in our study, either because they were not part of the post COVID-19 condition registry or because they were less inclined to fill out the survey. Third, respondents at the six different time periods since acute infection were not completely comparable, as more recently infected respondents were younger, more often female, had a higher education level, less comorbidity and were less often overweight or obese. As some of these factors were associated with worse HRQoL and others with better HRQoL, these differences might have affected our results. Fourth, the influence of different COVID-19 virus variants was not taken into account in our study, although previous research suggests that the chronic burden might differ between variants (43). Fifth, not all assumptions of the linear regression analyses were met, as the residuals for the regression analysis with EQ-5D utility scores as outcome were not normally distributed. Therefore, the results of these analyses might be somewhat less definite, but still provide a good indication. Lastly, although we included a wide range of symptoms and outcomes, as the knowledge on post COVID-19 condition is rapidly developing, there are several aspects that, in hindsight, we would have added and that are relevant for future research. For example, recent studies suggest that a substantial number of patients experience postural orthostatic tachycardia syndrome (POTS), which can interfere with daily functioning and impair quality of life (52). In addition, treatment options for post COVID-19 condition were also not explored in this study, but are a crucial topic for future research, as we slowly gain a better understanding of this condition.

In conclusion, regardless of time since infection, respondents considered fatigue, sensory processing problems and concentration problems the most debilitating symptoms. They experienced a low HRQoL and severe fatigue, even more than two years after acute COVID-19 infection. Respondents 3–6 m post-infection had the worst health outcomes, whereas respondents 13–18 m post-infection had the best outcomes, indicating that, at least for a subgroup of patients, health status may improve over time. Additional studies with a longer follow-up are needed to fully clarify the natural history of post COVID-19 condition and to elucidate risk factors for a severe disease course.

## Data Availability

The original contributions presented in the study are included in the article/**Supplementary Materials**, further inquiries can be directed to the corresponding author.

## References

[1] World Health Organization. WHO Coronavirus (COVID-19) Dashboard (2022). Available at: https://covid19.who.int/ (Accessed December 14, 2022).

[2] Soriano JB Murthy S Marshall JC Relan P Diaz JV, on behalf of the WHO Clinical Case Definition Working Group on Post-COVID-19 Condition. A clinical case definition of post-COVID-19 condition by a delphi consensus. Lancet Infect Dis. (2022) 22(4):e102–7. 10.1016/S1473-3099(21)00703-9 34951953 PMC8691845

[3] Global Burden of Disease Long CC, Wulf Hanson S Abbafati C Aerts JG Al-Aly Z Ashbaugh C Estimated global proportions of individuals with persistent fatigue, cognitive, and respiratory symptom clusters following symptomatic COVID-19 in 2020 and 2021. JAMA. (2022) 328(16):1604–15. 10.1001/jama.2022.18931 36215063 PMC9552043

[4] Ballering AV van Zon SKR Olde Hartman TC Rosmalen JGM, Lifelines Corona Research I. Persistence of somatic symptoms after COVID-19 in The Netherlands: an observational cohort study. Lancet. (2022) 400(10350):452–61. 10.1016/S0140-6736(22)01214-4 35934007 PMC9352274

[5] Phillips S Williams MA. Confronting our next national health disaster—long-haul COVID. N Engl J Med. (2021) 385(7):577–9. 10.1056/NEJMp2109285 34192429

[6] Yong SJ. Long COVID or post-COVID-19 syndrome: putative pathophysiology, risk factors, and treatments. Infect Dis (Lond). (2021) 53(10):737–54. 10.1080/23744235.2021.1924397 34024217 PMC8146298

[7] Crook H Raza S Nowell J Young M Edison P. Long COVID-mechanisms, risk factors, and management. Br Med J. (2021) 374:n1648. 10.1136/bmj.n1648 34312178

[8] Nasserie T Hittle M Goodman SN. Assessment of the frequency and variety of persistent symptoms among patients with COVID-19: a systematic review. JAMA Netw Open. (2021) 4(5):e2111417. 10.1001/jamanetworkopen.2021.11417 34037731 PMC8155823

[9] Fernández-de-Las-Peñas C Palacios-Ceña D Gómez-Mayordomo V Florencio LL Cuadrado ML Plaza-Manzano G Prevalence of post-COVID-19 symptoms in hospitalized and non-hospitalized COVID-19 survivors: a systematic review and meta-analysis. Eur J Intern Med. (2021) 92:55–70. 10.1016/j.ejim.2021.06.009 34167876 PMC8206636

[10] Han Q Zheng B Daines L Sheikh A. Long-Term sequelae of COVID-19: a systematic review and meta-analysis of one-year follow-up studies on post-COVID symptoms. Pathogens. (2022) 11(2):269. 10.3390/pathogens11020269 PMC8875269 35215212

[11] Fernández-de-Las-Peñas C Rodríguez-Jiménez J Cancela-Cilleruelo I Guerrero-Peral A Martín-Guerrero JD García-Azorín D Post-COVID-19 symptoms 2 years after SARS-CoV-2 infection among hospitalized vs nonhospitalized patients. JAMA Netw Open. (2022) 5(11):e2242106. 10.1001/jamanetworkopen.2022.42106 36378309 PMC9667330

[12] Taquet M Geddes JR Husain M Luciano S Harrison PJ. 6-month Neurological and psychiatric outcomes in 236 379 survivors of COVID-19: a retrospective cohort study using electronic health records. Lancet Psychiatry. (2021) 8(5):416–27. 10.1016/S2215-0366(21)00084-5 33836148 PMC8023694

[13] Malik P Patel K Pinto C Jaiswal R Tirupathi R Pillai S Post-acute COVID-19 syndrome (PCS) and health-related quality of life (HRQoL)-A systematic review and meta-analysis. J Med Virol. (2022) 94(1):253–62. 10.1002/jmv.27309 34463956 PMC8662132

[14] Davis HE Assaf GS McCorkell L Wei H Low RJ Re'em Y Characterizing long COVID in an international cohort: 7 months of symptoms and their impact. EClinicalMedicine. (2021) 38:101019. 10.1016/j.eclinm.2021.101019 34308300 PMC8280690

[15] Munblit D Nicholson TR Needham DM Seylanova N Parr C Chen J Studying the post-COVID-19 condition: research challenges, strategies, and importance of core outcome set development. BMC Med. (2022) 20(1):50. 10.1186/s12916-021-02222-y 35114994 PMC8813480

[16] Alkodaymi MS Omrani OA Fawzy NA Shaar BA Almamlouk R Riaz M Prevalence of post-acute COVID-19 syndrome symptoms at different follow-up periods: a systematic review and meta-analysis. Clin Microbiol Infect. (2022) 28(5):657–66. 10.1016/j.cmi.2022.01.014 35124265 PMC8812092

[17] Lancet T. Understanding long COVID: a modern medical challenge. Lancet. (2021) 398(10302):725. 10.1016/S0140-6736(21)01900-0 34454656 PMC8389978

[18] Statistics Netherlands (CBS). Opleidingsniveau. Available at: https://www.cbs.nl/nl-nl/nieuws/2019/33/verschil-levensverwachting-hoog-en-laagopgeleid-groeit/opleidingsniveau (Accessed December 14, 2022).

[19] WHO. Obesity: preventing and managing the global epidemic. Report of a WHO consultation. World Health Organ Tech Rep Ser. (2000) 894(i–xii):1–253. 11234459

[20] Statistics Netherlands (CBS). CBS: Permanent onderzoek leefsituatie. Voorburg/Heerlen: Statistics Netherlands (CBS) (2003).

[21] Tong A Baumgart A Evangelidis N Viecelli AK Carter SA Azevedo LC Core outcome measures for trials in people with coronavirus disease 2019: respiratory failure, multiorgan failure, shortness of breath, and recovery. Crit Care Med. (2021) 49(3):503–16. 10.1097/CCM.0000000000004817 33400475 PMC7892260

[22] Munblit D Nicholson T Akrami A Apfelbacher C Chen J De Groote W A core outcome set for post-COVID-19 condition in adults for use in clinical practice and research: an international delphi consensus study. Lancet Respir Med. (2022) 10(7):715–24. 10.1016/S2213-2600(22)00169-2 35714658 PMC9197249

[23] Herdman M Gudex C Lloyd A Janssen M Kind P Parkin D Development and preliminary testing of the new five-level version of EQ-5D (EQ-5D-5l). Qual Life Res. (2011) 20(10):1727–36. 10.1007/s11136-011-9903-x 21479777 PMC3220807

[24] Versteegh MM Vermeuelen KM Evers SMAA de Wit GA Prenger R Stolk EA. Dutch Tariff for the five-level version of EQ-5D. Value Health. (2016) 19(4):343–52. 10.1016/j.jval.2016.01.003 27325326

[25] Vercoulen J Alberts M Bleijenberg G. De checklist individuele spankracht (CIS). Gedragstherapie. (1999) 32(131):6.

[26] Worm-Smeitink M Gielissen M Bloot L van Laarhoven HWM van Engelen BGM van Riel P The assessment of fatigue: psychometric qualities and norms for the checklist individual strength. J Psychosom Res. (2017) 98:40–6. 10.1016/j.jpsychores.2017.05.007 28554371

[27] Carruthers BM van de Sande MI De Meirleir KL Klimas NG Broderick G Mitchell T Myalgic encephalomyelitis: international consensus criteria. J Intern Med. (2011) 270(4):327–38. 10.1111/j.1365-2796.2011.02428.x 21777306 PMC3427890

[28] Plummer F Manea L Trepel D McMillan D. Screening for anxiety disorders with the GAD-7 and GAD-2: a systematic review and diagnostic metaanalysis. Gen Hosp Psychiatry. (2016) 39:24–31. 10.1016/j.genhosppsych.2015.11.005 26719105

[29] Levis B Sun Y He C Wu Y Krishnan A Bhandari PM Accuracy of the PHQ-2 alone and in combination with the PHQ-9 for screening to detect Major depression: systematic review and meta-analysis. JAMA. (2020) 323(22):2290–300. 10.1001/jama.2020.6504 32515813 PMC7284301

[30] Yang T Yan MZ Li X Lau EHY. Sequelae of COVID-19 among previously hospitalized patients up to 1 year after discharge: a systematic review and meta-analysis. Infection. (2022) 50(5):1067–109. 10.1007/s15010-022-01862-3 35750943 PMC9244338

[31] Tran VT Porcher R Pane I Ravaud P. Course of post COVID-19 disease symptoms over time in the ComPaRe long COVID prospective e-cohort. Nat Commun. (2022) 13(1):1812. 10.1038/s41467-022-29513-z 35383197 PMC8983754

[32] Vatcheva KP Lee M McCormick JB Rahbar MH. Multicollinearity in regression analyses conducted in epidemiologic studies. Epidemiology (Sunnyvale). (2016) 6(2):227. 10.4172/2161-1165.1000227 27274911 PMC4888898

[33] Peter RS Nieters A Kräusslich HG Brockmann SO Göpel S Kindle G Post-acute sequelae of COVID-19 six to 12 months after infection: population based study. Br Med J. (2022) 379:e071050. 10.1136/bmj-2022-071050 36229057 PMC9557001

[34] Huang L Li X Gu X Zhang H Ren L Guo L Health outcomes in people 2 years after surviving hospitalisation with COVID-19: a longitudinal cohort study. Lancet Respir Med. (2022) 10(9):863–76. 10.1016/S2213-2600(22)00126-6 35568052 PMC9094732

[35] Bek LM Berentschot JC Heijenbrok-Kal MH Huijts S van Genderen ME Vlake JH Symptoms persisting after hospitalisation for COVID-19: 12 months interim results of the CO-FLOW study. ERJ Open Res. (2022) 8(4):355. 10.1183/23120541.00355-2022 36284829 PMC9421428

[36] de Ridder D Geenen R Kuijer R van Middendorp H. Psychological adjustment to chronic disease. Lancet. (2008) 372(9634):246–55. 10.1016/S0140-6736(08)61078-8 18640461

[37] Szende A Janssen B Cabases J. Self-reported population health: An international perspective based on EQ-5D. (2014). 29787044

[38] Van Herck M Goërtz YMJ Houben-Wilke S Machado FVC Meys R Delbressine JM Severe fatigue in long COVID: web-based quantitative follow-up study in members of online long COVID support groups. J Med Internet Res. (2021) 23(9):e30274. 10.2196/30274 34494964 PMC8457337

[39] Twomey R DeMars J Franklin K Culos-Reed SN Weatherald J Wrightson JG. Chronic fatigue and postexertional malaise in people living with long COVID: an observational study. Phys Ther. (2022) 102(4):pzac005. 10.1093/ptj/pzac005 35079817 PMC9383197

[40] Statistics Netherlands (CBS). Risico op angststoornis of depressie onder jongvolwassenen toegenomen (2021). Available at: https://www.cbs.nl/nl-nl/nieuws/2021/26/risico-op-angststoornis-of-depressie-onder-jongvolwassenen-toegenomen (Accessed December 14, 2022).

[41] Schou TM Joca S Wegener G Bay-Richter C. Psychiatric and neuropsychiatric sequelae of COVID-19—a systematic review. Brain Behav Immun. (2021) 97:328–48. 10.1016/j.bbi.2021.07.018 34339806 PMC8363196

[42] Stanton AL Revenson TA Tennen H. Health psychology: psychological adjustment to chronic disease. Annu Rev Psychol. (2007) 58:565–92. 10.1146/annurev.psych.58.110405.085615 16930096

[43] Magnusson K Kristoffersen DT Dell'Isola A Kiadaliri A Turkiewicz A Runhaar J Post-COVID medical complaints following infection with SARS-CoV-2 omicron vs Delta variants. Nat Commun. (2022) 13(1):7363. 10.1038/s41467-022-35240-2 36450749 PMC9709355

[44] Fernández-de-Las-Peñas C Notarte KI Peligro PJ Velasco JV Ocampo MJ Henry BM Long-COVID symptoms in individuals infected with different SARS-CoV-2 variants of concern: a systematic review of the literature. Viruses. (2022) 14(12):2629. 10.3390/v14122629 PMC9785120 36560633

[45] Notarte KI Catahay JA Velasco JV Pastrana A Ver AT Pangilinan FC Impact of COVID-19 vaccination on the risk of developing long-COVID and on existing long-COVID symptoms: a systematic review. EClinicalMedicine. (2022) 53:101624. 10.1016/j.eclinm.2022.101624 36051247 PMC9417563

[46] Coretti S Ruggeri M McNamee P. The minimum clinically important difference for EQ-5D index: a critical review. Expert Rev Pharmacoecon Outcomes Res. (2014) 14(2):221–33. 10.1586/14737167.2014.894462 24625040

[47] Subramanian A Nirantharakumar K Hughes S Myles P Williams T Gokhale KM Symptoms and risk factors for long COVID in non-hospitalized adults. Nat Med. (2022) 28(8):1706–14. 10.1038/s41591-022-01909-w 35879616 PMC9388369

[48] Wallenburg I Helderman JK Jeurissen P Bal R. Unmasking a health care system: the Dutch policy response to the COVID-19 crisis. Health Econ Policy Law. (2022) 17(1):27–36. 10.1017/S1744133121000128 33663625 PMC8007948

[49] Berentschot JC Heijenbrok-Kal MH Bek LM Huijts SM van Bommel J van Genderen ME Physical recovery across care pathways up to 12 months after hospitalization for COVID-19: a multicenter prospective cohort study (CO-FLOW). Lancet Reg Health Eur. (2022) 22:100485. 10.1016/j.lanepe.2022.100485 36039177 PMC9402257

[50] Strom JL Egede LE. The impact of social support on outcomes in adult patients with type 2 diabetes: a systematic review. Curr Diab Rep. (2012) 12(6):769–81. 10.1007/s11892-012-0317-0 22949135 PMC3490012

[51] Fincham JE. Response rates and responsiveness for surveys, standards, and the journal. Am J Pharm Educ. (2008) 72(2):43. 10.5688/aj720243 18483608 PMC2384218

[52] Ormiston CK Świątkiewicz I Taub PR. Postural orthostatic tachycardia syndrome as a sequela of COVID-19. Heart Rhythm. (2022) 19(11):1880–9. 10.1016/j.hrthm.2022.07.014 35853576 PMC9287587

