# Supplementary material for: The prolonged impact of COVID-19 on symptoms, health-related quality of life, fatigue and mental well-being: a cross-sectional study
Source: Front Epidemiol. 2023 Jun 22;3:1144707. doi: 10.3389/fepid.2023.1144707 (PMC10911032; doi:10.3389/fepid.2023.1144707)
Supplement: Supplementary file 1 [file Datasheet1.docx]

***Supplementary Material***

**The prolonged impact of COVID-19 on symptoms, health-related quality of life, fatigue and mental well-being: a cross-sectional study**

**Iris M. Brus*, Inge Spronk, Juanita A. Haagsma, Annemieke de Groot, Peter Tieleman, Sara Biere-Rafi and Suzanne Polinder**

*** Correspondence:** Corresponding Author: i.brus@erasmusmc.nl

**Table S1. Characteristics of total population (n=10,194)**

|  | *Median (IQR)* |
| --- | --- |
| Age in years | 49.0 (16.0) |
| Age in categories | ***N (%)*** |
| 18-24 years | 257 (2.5) |
| 25-34 years | 1,373 (13.5) |
| 35-44 years | 2,202 (21.6) |
| 45-54 years | 3,251 (31.9) |
| 55-64 years | 2,627 (25.8) |
| 65-74 years | 419 (4.1) |
| 75-88 years | 65 (0.6) |
| Gender |  |
| Male | 2,434 (23.9) |
| Female | 7.733 (75.9) |
| Other | 18 (0.2) |
| Rather not disclose | 9 (0.1) |
| Level of education |  |
| Low | 1,241 (12.2) |
| Middle | 3,504 (34.4) |
| High | 5,427 (53.2) |
| Unknown | 22 (0.2) |
| Occupational status before COVID-19 |  |
| Paid work | 9,306 (91.3) |
| Unpaid work | 204 (2.0) |
| No work | 684 (6.7) |
| Married/living with significant other |  |
| Yes | 7,336 (72.0) |
| No | 2,839 (27.8) |
| Unknown | 19 (0.2) |
| Comorbidity |  |
| No comorbidity | 5,373 (52.7) |
| Comorbidity | 4,821 (47.3) |
| BMI |  |
| Underweight (<18,5 kg/m^2^) | 143 (1.4) |
| Normal weight (18,5-25 kg/m^2^) | 4,148 (40.7) |
| Overweight (25-30 kg/m^2^) | 3,496 (34.3) |
| Obese (>30 kg/m^2^) | 2,400 (23.5) |
| Unknown | 7 (0.1) |
| COVID-19 diagnosis |  |
| Confirmed with test | 8,841 (86.7) |
| Not confirmed with test | 1,353 (13.3) |
| Number of COVID-19 infections |  |
| 1 infection | 7,690 (75.4) |
| 2 or more infections | 2,504 (24.6) |
| Hospital admission |  |
| Yes | 848 (8.3) |
| No | 9,346 (91.7) |
| Intensive care admission |  |
| Yes | 229 (2.2) |
| No | 9,975 (97.8) |
| Vaccination status |  |
| Vaccinated | 9.389 (92.1) |
| Not vaccinated | 706 (6.9) |
| Rather not disclose | 99 (1.0) |

**Table S2. Most debilitating symptoms per time period.**

This table shows the proportion of respondents that considered symptoms most debilitating, presented for respondents at different time periods since acute infection. *Statistically significant differences (p<0.05) between groups based on time since acute COVID-19 infection.

|  | 3-6 months | 7-9 months | 10-12 months | 13-18 months | 19-24 months | >24 months |
| --- | --- | --- | --- | --- | --- | --- |
| Fatigue* | 74.5% | 74.2% | 70.3% | 69.2% | 70.0% | 69.8% |
| Sensory processing problems* | 56.1% | 55.3% | 54.1% | 49.9% | 45.1% | 43.6% |
| Concentration problems* | 45.7% | 49.1% | 46.6% | 45.2% | 41.8% | 43.8% |
| Shortness of breath with exertion* | 32.9% | 31.8% | 33.8% | 34.0% | 38.0% | 34.3% |
| Decreased physical condition | 26.7% | 24.2% | 27.0% | 27.9% | 28.9% | 26.2% |
| Headache* | 32.3% | 28.9% | 27.6% | 26.6% | 21.3% | 22.2% |
| Memory problems* | 23.2% | 24.7% | 25.8% | 25.8% | 24.3% | 30.0% |
| Sleeping problems | 21.6% | 20.2% | 21.4% | 19.6% | 20.7% | 20.3% |
| Word finding problems | 13.7% | 15.5% | 13.2% | 14.8% | 14.2% | 13.9% |
| Heart palpitations* | 13.3% | 10.6% | 12.1% | 10.2% | 10.6% | 12.0% |
| Muscle soreness* | 9.2% | 9.9% | 9.2% | 11.6% | 11.8% | 11.2% |
| Irritability* | 11.4% | 11.2% | 12.2% | 9.9% | 7.3% | 10.1% |
| Pain in extremities* | 9.1% | 6.6% | 9.5% | 8.7% | 11.8% | 13.5% |
| Joint pain* | 6.6% | 8.0% | 8.0% | 9.0% | 11.3% | 11.2% |
| Dizziness* | 9.6% | 10.2% | 9.3% | 8.1% | 7.7% | 9.7% |
| Muscle weakness | 7.4% | 7.8% | 6.5% | 6.8% | 8.1% | 9.1% |
| Chest pain* | 8.0% | 5.7% | 6.9% | 6.1% | 8.9% | 6.8% |
| Depressive feelings* | 8.5% | 6.7% | 6.9% | 7.5% | 5.9% | 5.9% |
| Shortness of breath at rest* | 7.7% | 5.7% | 5.0% | 5.0% | 7.2% | 9.1% |
| Tingling in extremities | 5.0% | 4.5% | 4.6% | 5.7% | 5.7% | 4.9% |
| Anxiety | 5.6% | 4.5% | 4.4% | 4.3% | 4.4% | 3.4% |
| Loss of smell* | 2.5% | 4.1% | 6.0% | 5.5% | 3.9% | 2.8% |
| Cough* | 3.2% | 3.9% | 2.7% | 4.2% | 5.5% | 4.6% |
| Loss of taste* | 2.0% | 3.1% | 3.7% | 3.8% | 2.6% | 1.1% |
| Nausea | 3.0% | 2.5% | 2.8% | 2.7% | 3.4% | 2.8% |
| Skin problems* | 1.3% | 2.0% | 2.7% | 2.9% | 2.8% | 3.6% |
| Coughing up mucus* | 1.5% | 2.1% | 2.6% | 2.0% | 3.0% | 2.5% |
| Menstrual problems** | 1.4% | 2.0% | 2.5% | 2.6% | 2.7% | 2.2% |
| Sore throat* | 3.0% | 2.2% | 2.2% | 3.2% | 4.7% | 3.6% |
| Confusion | 2.8% | 2.4% | 1.6% | 1.8% | 1.7% | 1.9% |
| Diarrhea* | 1.5% | 1.4% | 1.4% | 1.5% | 2.3% | 3.0% |
| Fever* | 2.1% | 1.3% | 0.7% | 1.5% | 2.2% | 2.3% |
| Hair loss* | 2.1% | 0.8% | 0.7% | 1.1% | 1.5% | 0.9% |
| Decreased appetite | 1.4% | 1.5% | 1.0% | 1.0% | 1.2% | 1.9% |

**Menstrual problems are calculated for females aged 18-51 years old (n=4.212).

**Table S3. Health outcomes per time period.**

This table shows the health-related quality of life (EQ-5D-5L utility score and EQ VAS), proportion with a score indicative of anxiety disorder (GAD-2), proportion with a score indicative of depressive disorder (PHQ-2), energy level compare to before COVID-19, and fatigue level (CIS sum score and subscales) of respondents, presented for respondents at different time periods since acute infection.

|  |  | 3-6 months | 7-9 months | 10-12 months | 13-18 months | 19-24 months | >24 months | p-value |
| --- | --- | --- | --- | --- | --- | --- | --- | --- |
|  |  |  |  |  |  |  |  |  |
| EQ-5D-5L  utility score | Mean (SD) | 0.53 (0.25) | 0.56 (0.25) | 0.58 (0.25) | 0.58 (0.26) | 0.55 (0.27) | 0.52 (0.28) |  |
|  | Median (IQR) | 0.59 (0.32) | 0.62 (0.32) | 0.63 (0.31) | 0.65 (0.33) | 0.63 (0.38) | 0.60 (0.42) | <0.001 |
|  | Min.-max. | -0.34-1.00 | -0.34-1.00 | -0.35-1.00 | -0.41-1.00 | -0.41-1.00 | -0.34-1.00 |  |
| EQ-VAS | Mean (SD) | 44.4 (17.1) | 46.6 (17.6) | 49.9 (17.4) | 50.2 (18.6) | 49.6 (19.2) | 47.2 (18.7) |  |
|  | Median (IQR) | 44.0 (30.0) | 45.0 (29.0) | 50.0 (23.0) | 51.0 (29.0) | 50.0 (29.0) | 46.0 (26.0) | 0.000 |
|  | Min.-max. | 0-100 | 0-100 | 0-95 | 0-100 | 0-100 | 0-90 |  |
| GAD-2, cut-off ≥3 | n (%) | 26.2% | 28.7% | 24.7% | 23.7% | 22.2% | 24.9% | <0.001 |
| PHQ-2, cut-off ≥3 | n (%) | 32.4% | 32.1% | 27.0% | 27.6% | 25.0% | 29.2% | <0.001 |
| Energy level | Mean (SD) | 38.5 (19.2) | 43.7 (20.6) | 47.5 (20.8) | 49.3 (21.8) | 48.5 (22.7) | 43.6 (21.0) |  |
|  | Median (IQR) | 40.0 (25.0) | 40.0 (30.0) | 50.0 (31.3) | 50.0 (35.0) | 50.0 (35.0) | 40.0 (30.0) | 0.000 |
|  | Min.-max. | 0-100 | 0-100 | 2-100 | 0-100 | 0-100 | 0-100 |  |
| CIS sum score | Mean (SD) | 107.3 (17.7) | 105.7 (19.3) | 103.6 (20.1) | 103.1 (20.8) | 103.4 (20.9) | 105.2 (18.8) |  |
|  | Median (IQR) | 110.0 (23.0) | 108.0 (23.0) | 106.0 (25.0) | 106.0 (26.0) | 107.0 (26.0) | 108.0 (25.0) | <0.001 |
|  | Min.-max. | 20-140 | 24-140 | 20-140 | 20-140 | 20-140 | 29-140 |  |
| *CIS subscales* |  |  |  |  |  |  |  |  |
| Severity | Mean (SD) | 47.5 (8.2) | 46.5 (8.8) | 45.4 (9.3) | 45.5 (9.5) | 45.8 (9.5) | 46.7 (8.8) |  |
|  | Median (IQR) | 50.0 (11.0) | 49.0 (12.0) | 47.0 (12.0) | 48.0 (13.0) | 48.0 (12.0) | 49.0 (12.0) | <0.001 |
|  | Min.-max. | 8-56 | 11-56 | 8-56 | 8-56 | 8-56 | 13-56 |  |
| Concentration | Mean (SD) | 26.4 (6.5) | 26.2 (6.6) | 25.7 (6.7) | 25.3 (6.9) | 25.2 (6.8) | 25.8 (6.4) |  |
|  | Median (IQR) | 27.0 (8.0) | 27.0 (9.0) | 27.0 (9.0) | 26.0 (9.0) | 26.0 (9.0) | 27.0 (9.0) | <0.001 |
|  | Min.-max. | 5-35 | 5-35 | 5-35 | 5-35 | 5-35 | 5-35 |  |
| Motivation | Mean (SD) | 17.0 (6.0) | 17.1 (5.9) | 17.2 (5.9) | 17.2 (5.9) | 17.1 (6.0) | 17.1 (5.9) |  |
|  | Median (IQR) | 17.0 (10.0) | 17.0 (9.0) | 17.0 (9.0) | 18.0 (9.0) | 17.0 (9.0) | 17.0 (9.0) | 0.771 |
|  | Min.-max. | 4-28 | 4-28 | 4-28 | 4-28 | 4-28 | 4-28 |  |
| Physical activity | Mean (SD) | 16.4 (4.1) | 15.8 (4.4) | 15.3 (4.4) | 15.1 (4.6) | 15.2 (4.6) | 15.6 (4.4) |  |
|  | Median (IQR) | 17.0 (6.0) | 17.0 (6.0) | 16.0 (7.0) | 16.0 (7.0) | 16.0 (7.0) | 16.0 (6.0) | 0.000 |
|  | Min.-max. | 3-21 | 3-21 | 3-21 | 3-21 | 3-21 | 3-21 |  |

**Figure S1. EQ-5D dimensions per time period.**

This figure shows the proportion of respondents with problems at each EQ-5D dimension, presented for respondents at different time periods since acute infection.

**Figure S2. Fatigue (CIS) subscales per time period.**

This figure shows the median fatigue (CIS) subscale scores, presented for respodents at different time periods since acute infection. Subscale scores were transformed to a 0-100 scale, with higher scores indicating less fatigue, in order to be comparable.

**Table S4. Univariate analyses EQ-5D utility score.**

|  | 3-6 months | | 7-9 months | | 10-12 months | | 13-18 months | | 19-24 months | | >24 months | |
| --- | --- | --- | --- | --- | --- | --- | --- | --- | --- | --- | --- | --- |
|  | *n=1,672* | | *n=1,183* | | *n=1,333* | | *n=2,745* | | *n=2,574* | | *n=517* | |
|  | **B** | **p-value** | **B** | **p-value** | **B** | **p-value** | **B** | **p-value** | **B** | **p-value** | **B** | **p-value** |
| Age |  |  |  |  |  |  |  |  |  |  |  |  |
| 18-24 years | -7.546 | 0.028 | -6.051 | 0.114 | -0.611 | 0.884 | -5.123 | 0.111 | -11.966 | 0.009 | 0.149 | 0.987 |
| 25-34 years | -4.321 | 0.006 | -2.198 | 0.258 | -0.994 | 0.609 | -1.327 | 0.376 | -3.533 | 0.057 | -3.533 | 0.370 |
| 35-44 years | -0.059 | 0.966 | -3.413 | 0.049 | -0.372 | 0.827 | 0.329 | 0.788 | -1.553 | 0.259 | -0.445 | 0.891 |
| 45-54 years (ref) |  |  |  |  |  |  |  |  |  |  |  |  |
| 55-64 years | 2.861 | 0.062 | -0.530 | 0.770 | 0.321 | 0.841 | -0.969 | 0.394 | 0.731 | 0.531 | 0.669 | 0.797 |
| 65-88 years | 2.107 | 0.522 | 8.790 | 0.045 | 0.816 | 0.814 | 4.215 | 0.096 | 5.799 | 0.007 | 11.071 | 0.008 |
| Gender |  |  |  |  |  |  |  |  |  |  |  |  |
| Male | 5.899 | <0.001 | 7.072 | <0.001 | 4.303 | 0.007 | 3.929 | <0.001 | 6.763 | <0.001 | 1.105 | 0.667 |
| Female (ref) |  |  |  |  |  |  |  |  |  |  |  |  |
| Level of education |  |  |  |  |  |  |  |  |  |  |  |  |
| Low | 0.359 | 0.865 | -2.267 | 0.314 | -7.398 | <0.001 | -6.443 | <0.001 | -4.499 | 0.006 | -7.888 | 0.016 |
| Middle | -3.514 | 0.007 | -4.727 | 0.003 | -2.364 | 0.107 | -1.761 | 0.090 | -2.283 | 0.043 | -1.152 | 0.656 |
| High (ref) |  |  |  |  |  |  |  |  |  |  |  |  |
| Married/living with significant other |  |  |  |  |  |  |  |  |  |  |  |  |
| Yes (ref) |  |  |  |  |  |  |  |  |  |  |  |  |
| No | -3.392 | 0.012 | -4.831 | 0.003 | -2.110 | 0.195 | -2.204 | 0.053 | -4.170 | <0.001 | -3.192 | 0.226 |
| Occupational status before COVID-19 |  |  |  |  |  |  |  |  |  |  |  |  |
| Paid work (ref) |  |  |  |  |  |  |  |  |  |  |  |  |
| No paid work | -9.512 | <0.001 | -5.971 | 0.041 | -12.749 | <0.001 | -9.459 | <0.001 | -5.450 | 0.002 | -6.681 | 0.072 |
| Comorbidity |  |  |  |  |  |  |  |  |  |  |  |  |
| No comorbidity (ref) |  |  |  |  |  |  |  |  |  |  |  |  |
| Comorbidity | -5.266 | <0.001 | -5.800 | <0.001 | -7.086 | <0.001 | -6.925 | <0.001 | -8.389 | <0.001 | -10.017 | <0.001 |
| Overweight |  |  |  |  |  |  |  |  |  |  |  |  |
| Yes (ref) |  |  |  |  |  |  |  |  |  |  |  |  |
| No | 1.664 | 0.171 | 0.270 | 0.855 | 1.123 | 0.431 | 1.897 | 0.063 | 4.258 | <0.001 | 9.479 | <0.001 |
| Hospital admission |  |  |  |  |  |  |  |  |  |  |  |  |
| Yes | -1.640 | 0.676 | -1.862 | 0.533 | -2.527 | 0.266 | -2.864 | 0.090 | -1.787 | 0.311 | 3.373 | 0.449 |
| No (ref) |  |  |  |  |  |  |  |  |  |  |  |  |
| Vaccination status |  |  |  |  |  |  |  |  |  |  |  |  |
| Vaccinated (ref) |  |  |  |  |  |  |  |  |  |  |  |  |
| Not vaccinated | 0.213 | 0.925 | -8.620 | 0.001 | -8.893 | <0.001 | -4.015 | 0.058 | -6.426 | 0.004 | -6.419 | 0.141 |

This table shows the results of the univariate linear regression analyses, presented for respondents at different time periods since acute infection. Respondents with missing data on gender, level of education, household situation (married/living with significant other), or BMI, were excluded from the regression analyses (n=170; 1.7%). EQ-5D utility scores were transformed to a 0-100 scale.

**Table S5. Univariate analyses EQ VAS.**

|  | 3-6 months | | 7-9 months | | 10-12 months | | 13-18 months | | 19-24 months | | >24 months | |
| --- | --- | --- | --- | --- | --- | --- | --- | --- | --- | --- | --- | --- |
|  | *n=1,672* | | *n=1,183* | | *n=1,333* | | *n=2,745* | | *n=2,574* | | *n=517* | |
|  | **B** | **p-value** | **B** | **p-value** | **B** | **p-value** | **B** | **p-value** | **B** | **p-value** | **B** | **p-value** |
| Age |  |  |  |  |  |  |  |  |  |  |  |  |
| 18-24 years | -1.318 | 0.577 | -1.383 | 0.605 | -1.675 | 0.561 | -5.616 | 0.014 | -8.955 | 0.006 | -7.728 | 0.219 |
| 25-34 years | -2.506 | 0.021 | -2.148 | 0.114 | -0.686 | 0.609 | -2.636 | 0.013 | -3.177 | 0.015 | -1.677 | 0.530 |
| 35-44 years | -0.987 | 0.298 | -2.141 | 0.077 | -1.367 | 0.244 | -0.579 | 0.506 | -1.822 | 0.059 | -0.543 | 0.805 |
| 45-54 years (ref) |  |  |  |  |  |  |  |  |  |  |  |  |
| 55-64 years | 2.294 | 0.030 | 0.572 | 0.652 | 2.149 | 0.051 | 1.015 | 0.210 | 1.496 | 0.068 | 2.505 | 0.155 |
| 65-88 years | 3.192 | 0.159 | 12.633 | <0.001 | 4.794 | 0.045 | 3.142 | 0.082 | 8.317 | <0.001 | 7.560 | 0.007 |
| Gender |  |  |  |  |  |  |  |  |  |  |  |  |
| Male | 3.576 | <0.001 | 2.663 | 0.026 | 2.173 | 0.048 | 2.357 | 0.005 | 4.315 | <0.001 | -0.041 | 0.981 |
| Female (ref) |  |  |  |  |  |  |  |  |  |  |  |  |
| Level of education |  |  |  |  |  |  |  |  |  |  |  |  |
| Low | 1.928 | 0.185 | -1.222 | 0.438 | -1.654 | 0.237 | -1.498 | 0.165 | -1.329 | 0.246 | -3.274 | 0.140 |
| Middle | -1.624 | 0.069 | -1.999 | 0.069 | -1.536 | 0.129 | -0.384 | 0.604 | -0.307 | 0.699 | 1.079 | 0.537 |
| High (ref) |  |  |  |  |  |  |  |  |  |  |  |  |
| Married/living with significant other |  |  |  |  |  |  |  |  |  |  |  |  |
| Yes (ref) |  |  |  |  |  |  |  |  |  |  |  |  |
| No | -1.817 | 0.049 | -1.652 | 0.149 | -1.502 | 0.182 | -2.145 | 0.008 | -2.915 | <0.001 | -1.218 | 0.495 |
| Occupational status before COVID-19 |  |  |  |  |  |  |  |  |  |  |  |  |
| Paid work (ref) |  |  |  |  |  |  |  |  |  |  |  |  |
| No paid work | -0.182 | 0.913 | 0.169 | 0.934 | -2.123 | 0.229 | -2.923 | 0.023 | -0.646 | 0.600 | -0.025 | 0.992 |
| Comorbidity |  |  |  |  |  |  |  |  |  |  |  |  |
| No comorbidity (ref) |  |  |  |  |  |  |  |  |  |  |  |  |
| Comorbidity | -3.209 | <0.001 | -1.991 | 0.052 | -3.496 | <0.001 | -3.161 | <0.001 | -4.128 | <0.001 | -4.648 | 0.005 |
| Overweight |  |  |  |  |  |  |  |  |  |  |  |  |
| Yes (ref) |  |  |  |  |  |  |  |  |  |  |  |  |
| No | -0.212 | 0.799 | 0.642 | 0.534 | 0.514 | 0.602 | -0.188 | 0.796 | 1.811 | 0.019 | 4.215 | 0.011 |
| Hospital admission |  |  |  |  |  |  |  |  |  |  |  |  |
| Yes | 5.875 | 0.030 | 2.243 | 0.283 | 2.039 | 0.193 | 0.554 | 0.646 | 1.765 | 0.154 | 5.582 | 0.063 |
| No (ref) |  |  |  |  |  |  |  |  |  |  |  |  |
| Vaccination status |  |  |  |  |  |  |  |  |  |  |  |  |
| Vaccinated (ref) |  |  |  |  |  |  |  |  |  |  |  |  |
| Not vaccinated | 1.644 | 0.290 | -2.559 | 0.170 | -5.591 | 0.001 | -3.107 | 0.039 | -5.676 | <0.001 | 0.016 | 0.996 |

This table shows the results of the univariate linear regression analyses, presented for respondents at different time periods since acute infection. Respondents with missing data on gender, level of education, household situation (married/living with significant other), or BMI were excluded from the regression analyses (n=170; 1.7%).
